# Positive experiences of healthcare professionals with a mainstreaming approach of germline genetic testing for women with ovarian cancer

**DOI:** 10.1007/s10689-021-00277-7

**Published:** 2021-10-07

**Authors:** Kyra Bokkers, Ronald P. Zweemer, Marco J. Koudijs, Sanne Stehouwer, Mary E. Velthuizen, Eveline M. A. Bleiker, Margreet G. E. M. Ausems

**Affiliations:** 1 Division Laboratories, Pharmacy and Biomedical Genetics, Department of Genetics, University Medical Center Utrecht, Heidelberglaan 100, 3584 CX Utrecht, The Netherlands; 2 Department of Gynecological Oncology, University Medical Center Utrecht, Heidelberglaan 100, 3584 CX Utrecht, The Netherlands; 3 Division of Psychosocial Research and Epidemiology, The Netherlands Cancer institute, Plesmanlaan 121, 1066 CX Amsterdam, The Netherlands; 4 Department of Clinical Genetics, Leiden University Medical Center, Albinusdreef 2, 2333 ZA Leiden, The Netherlands

**Keywords:** Epithelial ovarian cancer, Genetic counseling, *BRCA*, Mainstream genetic testing, Online training

## Abstract

According to current guidelines, all women with epithelial ovarian cancer are eligible for genetic testing for *BRCA* germline pathogenic variants. Unfortunately, not all affected women are tested. We evaluated the acceptability and feasibility for non-genetic healthcare professionals to incorporate germline genetic testing into their daily practice. We developed and implemented a mainstreaming pathway, including a training module, in collaboration with various healthcare professionals and patient organizations. Healthcare professionals from 4 different hospitals were invited to participate. After completing the training module, gynecologic oncologists, gynecologists with a subspecialty training in oncology, and nurse specialists discussed and ordered genetic testing themselves. They received a questionnaire before completing the training module and 6 months after working according to the new pathway. We assessed healthcare professionals’ attitudes, perceived knowledge, and self-efficacy, along with the feasibility of this new mainstream workflow in clinical practice, and evaluated the use and content of the training module. The participation rate for completing the training module was 90% (N = 19/21). At baseline and after 6 months, healthcare professionals had a positive attitude, high perceived knowledge and high self-efficacy toward discussing and ordering genetic testing. Knowledge had increased significantly after 6 months. The training module was rated with an average of 8.1 out of 10 and was considered useful. The majority of healthcare professionals (9/15) was able to discuss a genetic test in five to 10 min. After completion of a training module, non-genetic healthcare professionals feel motivated and competent to discuss and order genetic testing themselves.

**Supplementary Information:**

The online version contains supplementary material available at 10.1007/s10689-021-00277-7.

## Introduction

Epithelial ovarian cancer (EOC) is the most lethal type of gynecological cancer; around 185,000 women die of the disease every year [1]. Genetic testing is currently recommended for all women with EOC [2–4] because of the high prevalence of pathogenic *BRCA* germline variants, irrespective of age of diagnosis or family history [5, 6]. Furthermore, the testing indications have been expanded, since the results allow for individualized treatment options with PARP inhibitors in women with platinum-sensitive EOC who carry a pathogenic germline or somatic variant in a *BRCA* gene [3, 7].

If a genetic test reveals a germline pathogenic variant in a cancer predisposition gene such as *BRCA1* or *BRCA2*, family members also become eligible for a genetic test. Family members who carry the same pathogenic variant can take measures to prevent cancer, or diagnose it at an early stage [2, 4, 8].

Despite the importance of genetic testing for affected women and their family members, studies have shown that substantial numbers of eligible cancer patients are currently not tested [9, 10]. This has led to many initiatives to improve the uptake of genetic testing [11]. One of these initiatives is mainstreaming of genetic testing. With mainstreaming, germline genetic testing is incorporated into routine cancer care and is offered to patients by non-genetic healthcare professionals (HCP) treating them [12, 13].

Mainstream genetic testing initiatives for women with EOC have been successfully implemented in several countries, with positive experiences among patients and HCPs [12–19]. Although several of these initiatives included some form of education in genetic counseling, they did not evaluate HCPs’ experiences with these training modules [12–17, 19–23]. We consider it important to ensure that HCPs who are not formally trained in genetics and genetic counseling have sufficient knowledge and self-efficacy to discuss genetic testing before mainstream genetic testing is implemented into the routine care of women with EOC.

In the current study, we aimed to develop and implement a mainstreaming pathway for germline genetic testing in women with EOC, including an online training module for gynecologic oncologists, gynecologists with a subspecialty training in oncology, and nurse specialists. Our specific research objectives were: (1) to assess HCPs’ attitudes toward and knowledge of mainstream genetic testing, and their self-efficacy to discuss genetic testing before and 6 months after completion of a training module, (2) to have our training module evaluated by the users, and (3) to gain insight into the feasibility for HCPs to incorporate mainstream genetic testing into the routine care of women with EOC.

## Material and methods

### Development of the training module

We developed a concise online training module for all participating HCPs. The content of this training module was determined by our project team, which consisted of HCPs from the departments of genetics, gynecology, medical oncology and pathology involved in the care of women with EOC, and patient advocates. This resulted in four short (7 min each) educational films (see Supplementary file 1).

### Development and implementation of the care pathway for mainstream genetic testing

**Fig. 1:**
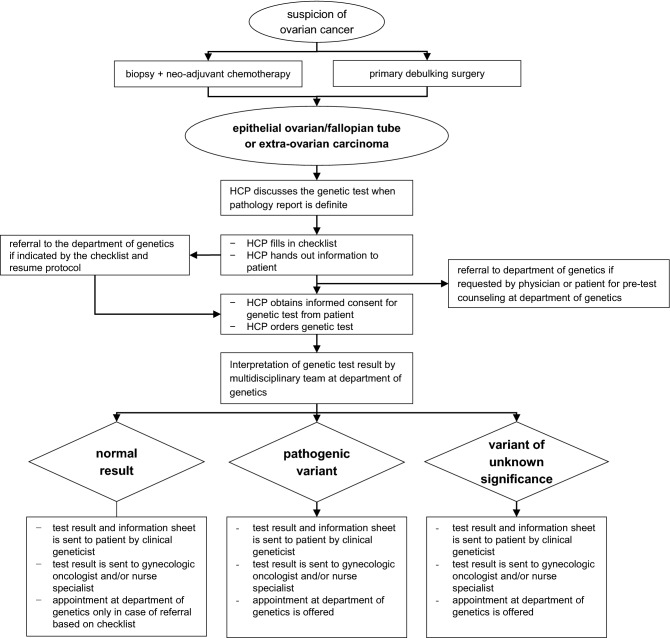
Flow-chart for mainstreaming pathway for healthcare professionals offering germline genetic testing to women with ovarian cancer

Our pathway for mainstream genetic testing was adapted from the workflow developed in the Mainstreaming Cancer Genetics Programme [13]. Flowchart shown in Fig. 1.

We organized a kick-off meeting in four hospitals in the Utrecht region to introduce the new workflow. These four hospitals are involved in the care of patients with ovarian cancer in this region. Gynecologic oncologists, gynecologists with a subspecialty training in oncology, and nurse specialists working in these hospitals were subsequently invited to complete the online training module in a personal electronic learning environment. After completion, HCPs received a manual with instructions and all necessary forms, including a patient information letter.

In our pathway, trained HCPs discussed the possibility of germline genetic testing (*BRCA1/2, RAD51C/D and BRIP1*) and the implications for family members with all newly diagnosed women with EOC (including fallopian tube and extra ovarian carcinomas) and women who had a personal history of EOC and had not been tested previously. In addition, HCPs completed a checklist (see Supplementary file 2) for every woman indicating whether she required additional counseling at the department of genetics after receiving their test result (e.g., indication for Lynch syndrome testing based on patient and/or family history or multiple family members with ovarian cancer implying preventive measures for female family members). If indicated by this checklist based on our national guidelines [2, 24], the HCP referred the patient to the department of genetics for further counseling.

After discussing the possibility of testing, written information about genetic testing was handed out to patients. If patients agreed to undergo genetic testing, they completed a written consent form and the test was ordered.

Patients received their test result in a letter from the department of genetics, along with general information about this result. A copy of this letter was sent to both the HCP who ordered the genetic test and the general practitioner. In the event of a positive test result, i.e., a pathogenic variant or variant of unknown significance, an appointment at the department of genetics was added to the letter. Patients with a negative test result received an invitation for an appointment only if indicated by the checklist.

This pathway was implemented in all four hospitals: implementation started in the first hospital in April 2018 and in the last one in June 2019 (one academic hospital and three non-academic teaching hospitals).

### Study procedure

We used a prospective follow-up design. The participating HCPs received two questionnaires. A first questionnaire (T0) had to be completed before starting the online training module and a second questionnaire (T1) was sent 6 months after implementing the new mainstreaming pathway for genetic testing. These questionnaires consisted of self-developed questions based on previous research by George et al. [13].

#### Attitude, perceived knowledge, self-efficacy, and knowledge

The T0 and T1 questionnaires consisted of 13 statements to assess HCPs’ attitudes (five statements), perceived knowledge (three statements), and self-efficacy (five statements) toward mainstream genetic testing [13]. The HCPs rated these statements using a 5-point Likert rating scale (from strongly disagree to strongly agree). In addition, this second questionnaire contained one extra question about attitudes toward mainstream genetic testing.

Both questionnaires contained the same five knowledge questions, including two statements, and three multiple choice questions. Every statement had three response-categories: true, false and ’I do not know’.

#### Evaluation of the training module

After each film, HCPs completed a self-developed questionnaire about the relevance of the discussed topics (5-point Likert scale from not useful at all to very useful) and their opinions regarding the duration of the films (5-point scale from far too long to far too short). Appreciation for each film was evaluated on a scale of one to 10.

At the end of the training module, we asked participants to assess the module as a whole, with appreciation on a scale of one to 10, and the usefulness, level of difficulty, duration, and their appreciation of the online format, using a 5-point Likert scale. In addition, they were asked whether they thought that important content was missing or whether they had ideas or advice on improving the training module.

After working according to this new workflow for 6 months, we asked the HCPs whether, in retrospect, they felt any information was missing from the training module.

#### Feasibility

We measured the feasibility of HCPs incorporating genetic testing into their routine work in relation to three outcomes: time investment, reasons for not discussing genetic testing, and how often additional appointments were needed to discuss and order genetic testing.

After 6 months, the HCPs estimated how much time they needed to discuss and order genetic testing (less than five minutes, 5–10 min, 10–20 min, more than 20 min). In addition, they were asked to rate whether this time investment was ‘as expected’ on a 5-point Likert rating scale (much worse than expected to much better than expected).

Both at baseline and after 6 months, the HCPs were asked to provide the two most important reasons for not discussing the possibility of genetic testing with all eligible patients.

After 6 months they estimated how often their patients needed an additional appointment to discuss genetic testing, and reported the most important reasons for such an appointment.

### Statistical Analyses

We used descriptive statistics to detail the characteristics of the HCPs, time investment, reasons for not discussing the possibility of genetic testing, and whether additional appointments were needed. We compared the characteristics of the HCPs in the T0 and T1 groups using the independent t-test for continuous variables and the chi-square test for categorical variables to determine whether the HCPs who filled in both questionnaires were representative of the entire group.

With paired analysis using the Wilcoxon signed-rank test we compared the total number of correct answers to the knowledge questions between T0 and T1, and all statements regarding attitude, perceived knowledge and self-efficacy between T0 and T1. A p-value < 0.05 was considered as statistically significant. Statistical analyses were performed using IBM SPSS statistics 25.0.0.2.

## Results

### Participation

Twenty-one HCPs received login codes to the online training module. Nineteen (90%) HCPs completed the entire training module. One HCP completed part of the online training module.

The first questionnaire was completed by 20 out of 21 HCPs (95%) from four hospitals. The second questionnaire was completed by 15 out of 17 HCPs. Two HCPs were not sent a second questionnaire because they had completed the online training module less than 6 months before the end of our study period.

The total group consisted of 20 HCPs. Their characteristics are presented in Table 1. There were no statistically significant differences in the characteristics of the 15 HCPs who filled in both questionnaires compared to the five HCPs who only completed the first questionnaire.

**Table 1:** Characteristics of participating HCPs

Characteristics of HCPs	Total group n = 20
Mean age (range)	47 (31–64)
Sex, n (%)	
Female	12 (60)
Male	8 (40)
Disciplines, n (%)	
Gynecologic oncologist	5 (25)
Gynecologist with a subspecialty training in oncology	7 (35)
Gynecologist in training	2 (10)
Nurse or nurse specialist (in training)	6 (30)
Hospital, n (%)	
Academic hospital	7^a^ (33.3)
Non-academic teaching hospital	14^a^ (66.7)

^a^One healthcare professional worked in both an academic and non-academic teaching hospital

### Attitude, perceived knowledge, self-efficacy, and knowledge

Table 2 shows the number of HCPs that ‘agreed’ or ‘strongly agreed’ with the statements regarding attitude, perceived knowledge, and self-efficacy toward mainstream genetic testing. Both at baseline and after 6 months, a majority of HCPs agreed (strongly) to most of these statements. Only for the statements about attitude and self-efficacy related to offering genetic testing directly after diagnosing ovarian cancer, the majority of HCPs neither agreed nor disagreed at both time points. With paired analysis, there were no significant differences between T0 and T1 for any of these statements. However, there seems to be a positive trend in the perceived knowledge of HCPs regarding the advantages and disadvantages of genetic testing (p = 0.058).

**Table 2:** Attitude, perceived knowledge, and self-efficacy of HCPs (N = 15) before (T0) and 6 months after completing the training module (T1)

Questions	T0 (strongly) agree n (%)	T1 (strongly) agree n (%)	p value
*Attitude*			
It is important for patients to have a choice whether or not to have a genetic test performed	14 (93.3)	13 (86.7)	ns
It is important to offer genetic testing immediately after diagnosing ovarian cancer	6 (40)	5 (33.3)	ns
It is important that all patients with ovarian cancer have access to genetic testing	15 (100)	15 (100)	ns
I am positive toward offering a genetic test myself	14 (93.3)	14 (93.3)	ns
It is important when discussing genetic testing to pay attention to the psychosocial consequences of genetic testing	14 (93.3)	14^a^ (100)	ns
Gynecologic oncologists, oncologists with a subspecialty training in oncology, and nurse specialists are capable of discussing and ordering genetic testing themselves after completing an online training module	na	13 (86.6)	ns
*Perceived knowledge*			
I understand the advantages and disadvantages of a genetic test	12 (80)	15 (100)	0.058
I understand the importance of genetic testing for patients with ovarian cancer	14 (93.3)	15 (100)	ns
I understand the importance of genetic testing for family members of patients with ovarian cancer	15 (100)	15 (100)	ns
*Self-efficacy*			
I am confident that I can discuss the advantages and disadvantages of a genetic test	15 (100)	13 (86.7)	ns
I am confident that I am able to discuss a genetic test with all patients with ovarian cancer directly after diagnosing ovarian cancer	8 (53.3)	7 (46.7)	ns
I am confident that I am able to order a genetic test myself	15 (100)	15 (100)	ns
I am confident that I am able to recognize psychosocial problems in patients and subsequently refer patients to a specialist social worker	15 (100)	14 (93.3)	ns
I am confident that I am able to explain what genetic testing in tumor tissue entails and what the differences are with genetic testing in blood samples	12 (80)	15 (100)	ns

The remaining HCPs either answered: ‘neither agree, nor disagree’, ‘disagree’, or ‘strongly disagree’

*na* not applicable, *ns* not significant

^a^One missing value

One HCP had a neutral attitude toward discussing and ordering genetic testing at baseline and thought that discussing and ordering genetic testing would be too time-consuming. The same HCP did have a positive attitude after 6 months. Another HCP had a positive attitude at baseline but a neutral attitude after 6 months; the new workflow was too time-consuming, this HCP felt insecure about their knowledge regarding genetic testing, and felt that clinical geneticists and genetic counselors had more experience and tools to discuss genetic testing.

Table 3 shows the knowledge questions and how many HCPs answered these questions correctly at baseline (before the online training module) and 6 months after implementing the new mainstreaming pathway. Paired analysis (between T0 and T1) for the total number of correct answers for all five questions were available for 14 HCPs. The total number of correct answers remained constant for seven HCPs after working for 6 months according to the new mainstreaming pathway, and improved for the other seven HCPs. The measured difference with paired analysis is statistically significant (p = 0.016).

**Table 3:** Knowledge of HCPs (N = 14) before (T0) and 6 months after completing the training module (T1)

Questions	T0 Correct answer N (%)	T1 Correct answer N (%)
What is the prevalence of *BRCA* mutations in patients with ovarian cancer?	3 (21.4)	9 (64.3)
Patients with ovarian cancer are eligible for genetic testing only when other family members have breast and/or ovarian cancer	14 (100)	14 (100)
A hereditary cause for ovarian cancer can be excluded if no mutation is found in one of the *BRCA* genes	13 (92.9)	13 (92.9)
What is the meaning of a *BRCA* mutation that is found with a tumor test only?	11 (78.6)	13 (92.9)
What is the meaning of a *BRCA* mutation that is found with a blood test only?	11 (78.6)	14 (100)

### Evaluation of the training module

The four individual films were ranked, with an average rating of between 7.9 and 8.1 out of 10. The majority (> 75%) of HCPs considered the duration of each individual film to be ‘exactly right’ and all discussed topics to be relevant.

The evaluation of the overall online training module is shown in Table 4. Immediately after completing the training module, two out of 19 HCPs mentioned that they missed information regarding the impact of genetic testing on insurance. After 6 months, two out of 15 HCPs mentioned that, in retrospect, they missed practical tips on how to order genetic testing. In addition, one HCP would have wanted to know the estimated time investment for discussing and ordering genetic testing.

**Table 4:** Evaluation of the overall online training module (n = 19 HCPs)

Average rating out of 10 (range)	8.1 (7–10)
Usefulness of online training module, n (%)	
(reasonably/very) useful	18 (94.7)
Not useful (at all)	1 (5.3)
Level of difficulty, n (%)	
(much) too high	0 (0)
Exactly right	16 (84.2)
(much) too low	3 (15.8)
Appreciation of online format, n (%)	
(very) pleasant	16 (84.2)
Fairly pleasant	3 (15.8)
Not pleasant (at all)	0 (0)
Duration of online training module, n (%)	
(much) too long	2 (10.5)
Exactly right	17 (89.5)
(much) too short	0 (0)

### Feasibility

HCPs were able to discuss a genetic test in five to 10 min (9/15) or 10 to 20 min (6/15). For 14 out of 15 HCPs this time investment was as expected or better than expected. Most HCPs were able to order the genetic test in less than 5 min (8/14), the remainder needed 5 to 10 min (n = 5) or 10 to 20 min (n = 1). For 13 out of 14 HCPs this time investment was as expected or better than expected.

**Fig. 2:**
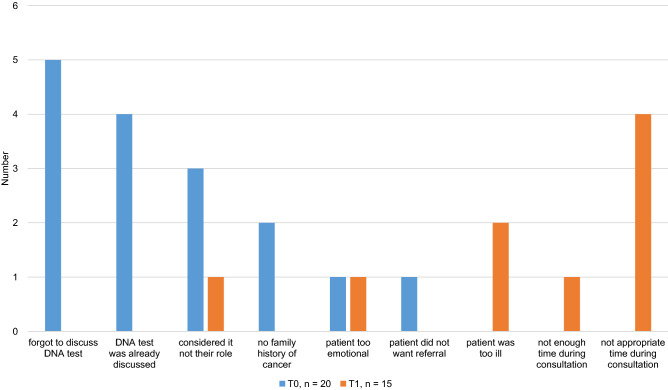
The main reasons for not discussing genetic testing before (T0) and 6 months after completing the training module (T1)

The main reasons for not discussing genetic testing differed between ‘forgotten’ (T0) and ‘no appropriate moment’ (T1) and are illustrated in Fig. 2.

Less than half (7/15) of the HCPs reported that they had scheduled an additional appointment to discuss genetic testing for at least one patient. Reasons for this were that patients needed reflection time to consider genetic testing (n = 6), that there was not enough time during the consultation (n = 4), and that discussing genetic testing would be too much information for the patient in one consultation (n = 4).

## Discussion

This study shows that gynecologic oncologists, gynecologists with a subspecialty training in oncology and nurse specialists feel motivated and competent to discuss and order germline genetic testing in women with EOC themselves. HCPs had a positive attitude, high self-efficacy, and high perceived knowledge both before and 6 months after working according to the new workflow. This high acceptability among HCPs is comparable to the results of other mainstreaming studies [12–14, 16, 17, 19]. Furthermore, 90% of the invited HCPs from four hospitals completed the training module and participated in our study.

We considered training an essential part of the implementation of our mainstream genetic testing pathway. Pre-test genetic counseling and informed consent are important, because the results of a genetic test can have serious implications for patients and family members [2, 4]. Training on genetic counseling should therefore also focus on the practical and emotional implications of a genetic test [25, 26]. We incorporated these aspects into our training module (see Supplementary file 1). Earlier research has shown that a majority of HCPs consider their knowledge about genetic testing to be inadequate [27]. A training intervention can be successful in increasing the perceived knowledge, self-efficacy and positive attitude of HCPs toward discussing and ordering genetic testing [14]. In our study, HCPs already had a positive attitude, high perceived knowledge, and high self-efficacy at baseline. This might be due to the close collaboration between the gynecology and genetics departments in our region. These outcomes persisted 6 months after completing our training module, which may be due to a ceiling effect. Importantly, after completing our online training module and with 6 months hands-on experience in discussing and ordering genetic testing themselves, the attitude remained positive, and perceived knowledge and self-efficacy remained high. We did see a significant increase in knowledge about genetic testing after 6 months. However, it should be noted that we asked a limited number of questions that do not cover all necessary aspects for pre-test counseling. The experiences of patients are the best indicator whether non-genetic HCPs are well equipped to perform pre-test counseling themselves.

The majority of HCPs take around 10 min to discuss and order genetic testing, which was ‘as expected’ or ‘better than expected’ for almost all of them. This time investment is comparable to the results from previous studies [14, 17] and seems to be acceptable to HCPs. Furthermore, 86% of HCPs agreed that, after completion of the training module, mainstream genetic testing should be incorporated into their routine work. This time investment is substantially shorter than the average time investment for traditional genetic counseling (on average 40 to 45 min) [28, 29]. For patients with EOC, an extended family history is not necessary to determine if the patient is eligible for genetic testing. We developed a short standardized checklist to evaluate family history in our mainstream model. In addition, we expect that non-genetic HCPs explain possible implications of a genetic test in a more general way.

After 6 months, the participants reported they no longer forgot to discuss genetic testing and their main reason for not discussing genetic testing was because they thought there was no appropriate moment during the consultation. In addition, about half of the HCPs felt confident discussing genetic testing with patients directly after diagnosing ovarian cancer. A standard moment to discuss and order genetic testing can further reduce the risk of HCPs forgetting to discuss it. However, it is important to take into consideration the timing of the different consultations and the amount of information that patients already receive after diagnosis. There are differences between hospitals, and adaptation of pathways or division of tasks in accordance with local workflows is often necessary. Our findings show that it is feasible for HCPs to incorporate germline genetic testing, including asking for patients’ written informed consent, into their daily work. Gleeson et al. showed that the most important barrier for non-genetic HCPs to continue with mainstream genetic testing was that they did not feel confident that there was a structured workflow, including collaboration with a department of genetics [14]. During our study period, this new workflow was already incorporated into standard care, and HCPs could discuss and order genetic testing for all women with EOC.

An advantage of the workflow that we implemented is that it can easily be adapted if gene panels change. In the course of our study, the ovarian cancer gene panel consisted of the five core genes (*BRCA1, BRCA2, BRIP1, RAD51C* and *RAD51D)*, and it is likely that the gene panel will be expanded with other cancer genes, such as *PALB2,* in the near future. Tumor testing can also be incorporated into our workflow. Tumor testing has the advantage that it can be used as a pre-test for germline genetic testing [30]. When a pathogenic variant is found in the tumor there is a 50% chance of the existence of a germline pathogenic variant, and patients and their family members should be prepared for this outcome. Therefore, adequate pre-test counseling and informed consent are equally important when discussing and ordering a tumor test first. Our training module covers the difference between germline and somatic variants. Therefore, after completing our training module, HCPs will be well equipped to first discuss and order a tumor test, and, if necessary, subsequently a germline test. An additional advantage of incorporating our workflow into a tumor first workflow is that germline testing can be offered directly to patients when a tumor test fails or cannot be performed.

Although our new workflow seemed feasible in this study setting, the financial consequences need to be taken into account. There should be adequate reimbursement for the extra time investment that HCPs need when discussing and ordering genetic testing. Future research should focus on the shift of responsibilities between the involved departments and the changes in financial sources.

A major strength of our study is that we developed our training module and workflow in collaboration with our project team consisting of multiple HCPs and two patient organizations. We could therefore identify barriers and facilitators for all involved parties. Other strengths are the before-and-after design to test the knowledge, attitude, and self-efficacy of HCPs, the inclusion of both academic and non-academic teaching hospitals, and the subsequent high participation rate of HCPs which improves the generalizability of our outcomes.

There are limitations for this study. Our study population was small, which makes it more difficult to observe significant effects, and we did not use standardized questionnaires to assess knowledge, attitude, and self-efficacy. To our knowledge, there are no suitable and validated questionnaires available to generate results that would answer our specific research questions. In addition, we only measured self-reported outcomes, and did not objectively measure skills. Therefore, the results of our study cannot easily be extrapolated to other non-genetic HCPs. Last, we did not compare our results to a control group of HCPs that did not receive any training in pre-test genetic counseling.

### Future research

For mainstream models to be successful and effective it is important that patients can make a well informed decision regarding genetic testing after pre-test counseling. So far, the experiences of patients with mainstream genetic testing have been investigated in multiple studies, but as far as we know there are no randomized trials. In addition, there is a lack of studies that focus on more quality of care outcomes [12, 13, 15, 17, 19]. In the future, patient experiences should be evaluated in more detail and should include not only satisfaction, but also outcomes that evaluate quality of care, such as patients’ understanding of received information, decisional conflict, and decision regret. In addition, it is important to consider alternative models that address the increasing demand for genetic testing, and to compare these alternative models, such as direct genetic testing models [31, 32], embedding genetic counsellors into oncology clinics [33, 34], and tumor-first genetic testing models [30].

## Conclusion

Preceded by an online training module, the implementation of a mainstreaming pathway for germline genetic testing in women with EOC seems feasible and acceptable for non-genetic HCPs.

## Supplementary Information

Below is the link to the electronic supplementary material.

Supplementary file1 (DOCX 20 kb)

Supplementary file2 (DOCX 23 kb)

## Data Availability

The datasets generated during and/or analyzed during the current study are available from the corresponding author on reasonable request.
