# Supplementary material for: Positive experiences of healthcare professionals with a mainstreaming approach of germline genetic testing for women with ovarian cancer
Source: Fam Cancer. 2021 Oct 7;21(3):295–304. doi: 10.1007/s10689-021-00277-7 (PMC9203381; doi:10.1007/s10689-021-00277-7)
Supplement: Supplementary file 1 — Supplementary file1 (DOCX 20 kb) [file 10689_2021_277_MOESM1_ESM.docx]

**Supplementary file 1 Supporting information regarding online training module**

The training module was evaluated and accredited by national accreditation bureaus of the scientific organizations for gynecologists and nurse specialists as part of the national CME accreditation.

Film 1: Mainstreaming of genetic testing in patients with cancer (duration: 7.5 minutes).

*This film contains the following elements:*

- Short introduction film with a clinical geneticist giving an explanation of the term mainstreaming of genetic testing and why this new workflow is important for patients with cancer.
- Slides with a voice over explaining:
  - How cancer develops and how hereditary and environmental factors play a role.
  - The difference between germline and somatic mutations.
  - The difference between genetic testing in blood and genetic testing in tumor tissue.
  - The importance of genetic testing in patients with cancer.
  - The difference between diagnostic and predictive genetic testing.
  - A summary of the above.

Film 2: *BRCA1* and *BRCA2*, genetic testing in ovarian cancer patients (duration: 5.5 minutes).

*This film contains the following elements:*

- Short introduction film with a clinical geneticist giving an overview of the content of the film.
- Slides with a voice over explaining:
  - The eligibility criteria for genetic testing in patients with ovarian cancer.
  - The prevalence of pathogenic variants in the *BRCA* genes and the difference in prevalence between the histologic subtypes.
  - Cancer risks associated with pathogenic variants in the *BRCA* genes.
  - Pattern of inheritance for pathogenic variants in the *BRCA* genes.
  - Screening recommendations for family members.
  - Possible changes in the future, for example the expected expansion of the gene panel and the role of tumor testing.
  - A summary of the above.

Film 3: The new workflow for genetic testing (duration: 7 minutes)

*This film contains the following elements:*

- Short introduction film with a clinical geneticist giving an overview of the content of the film.
- Sides with a voice over explaining:
  - The entire workflow (step-by-step).
  - How to fill out the checklist to determine if the patient is eligible for referral to the department of genetics.
  - How to fill out the informed consent form.

Film 4: Practical advice on how to discuss genetic testing (duration: 10 minutes)

*This film contains the following elements:*

- Short introduction film with a clinical geneticist giving an overview of the content of the film.
- Slides with a voice over explaining:
  - Communication about and timing of genetic testing.
  - Elements to discuss with a patient.
  - Possible outcomes of a genetic test and the implications of these outcomes.
- Film with:
  - A gynecologic oncologist and a patient. This shows a simulation consultation on how to discuss a genetic test, including:
    - Importance of genetic testing (possible implications for treatment options and risk of breast cancer).
    - Possible consequences for family members.
    - Procedure (blood test, time to result, and result send via letter).
  - A patient who is carrier of a pathogenic variant in the BRCA1 gene and currently using PARP inhibitors. She explains the impact that genetic testing had on her and her family and why genetic testing is important for every woman with ovarian cancer.
  - A specialized social worker. She explains what impact a pathogenic variant in a *BRCA* gene can have on patient and her family members, elaborates on the emotional consequences and explains the supportive role of a social worker in the process of genetic testing.
