# Supplementary material for: Positive experiences of healthcare professionals with a mainstreaming approach of germline genetic testing for women with ovarian cancer
Source: Fam Cancer. 2021 Oct 7;21(3):295–304. doi: 10.1007/s10689-021-00277-7 (PMC9203381; doi:10.1007/s10689-021-00277-7)
Supplement: Supplementary file 2 — Supplementary file2 (DOCX 23 kb) [file 10689_2021_277_MOESM2_ESM.docx]

**Supplementary file 2 checklist indicative for referral to genetics department**

**Checklist ‘referral to genetics department’**

yes no

Does your patient have:

- a first or second degree relative with ovarian cancer?
- a synchronous or metachronous Lynch syndrome associated carcinoma*?
- a first degree relative with endometrial or colon cancer, and diagnosed
  under the age of 50 years or the ovarian cancer diagnosed under the
  age of 50 years.
- 2 or more first or second degree relatives with a Lynch syndrome associated
  carcinoma*?
- (current or previous) breast cancer or ductal carcinoma in situ?

*Lynch syndrome associated carcinomas: colorectal carcinoma, endometrial carcinoma, stomach cancer, carcinoma of the small intestine, urinary tract carcinomas, ovarian cancer, pancreatic carcinoma, brain tumor, bile duct carcinoma and sebaceous gland carcinoma.

**Can 1 or more questions be answered with “yes”? 🡪 patient is eligible for referral to the genetics department**

**This checklist should be included in the patient file.**

Contact the genetics department easily when you have questions regarding this checklist or when you think the patient is eligible for referral and this is not reflected in the checklist. (original checklist includes telephone numbers)
